# Effects of Temporary Rearing on the Muscle Flavor Quality of *Chinemys reevesii*

**DOI:** 10.1002/fsn3.4728

**Published:** 2025-01-12

**Authors:** Xiaorong Lu, Yali Yu, Lixue Dong, Gang He, Lang Zhang, Tao Mao, Yun Liu, Yuntao Zhou, Li He

**Affiliations:** ^1^ Chinese Academy of Fishery Sciences Yangtze River Fisheries Research Institute Wuhan China; ^2^ Huazhong Agricultural University Wuhan China; ^3^ Jiangxi Institute of Fishery Sciences Nanchang China; ^4^ Hubei Institute for Drug Control Wuhan China

**Keywords:** *Chinemys reevesii*, flavor, temporary rearing, turtle, volatile odor compounds

## Abstract

*Chinemys reevesii* , a special economic aquaculture species in China, is valued highly for its medicinal and nutritional benefits. However, the muscle of farmed *C. reevesii* exhibits a strong off‐flavor, resulting in poor flavor quality. To enhance the flavor quality of the *C. reevesii* meat, this study examined the volatile compounds in *C. reevesii* muscle by establishing identification methods for these volatile odor compounds and comparing the differences between the two aquaculture modes. It also explored the impact of fasting temporary rearing (60 days) on the flavor quality of the *C. reevesii* meat. The results indicated that a combination of direct solvent extraction‐solvent‐assisted flavor evaporation (DSE‐SAFE), headspace‐solid phase microextraction (HS‐SPME), gas chromatography–mass spectrometry/olfactometry (GC–MS/O), and aroma recombination effectively simulated the odor profile of *C. reevesii* meat. The study identified the key volatile odor compounds in outdoor pond‐cultivated *C. reevesii* meat (OP), including hexanal, heptanal, 1‐octen‐3‐ol, octanal, nonanal, (E)‐2‐nonenal, decanal, (E)‐2‐decenal, (E,E)‐2,4‐decadienal, and dodecanal. In greenhouse‐cultivated *C. reevesii* meat (GH), key volatile odor compounds were hexanal, hexanol, heptanal, 1‐octen‐3‐ol, octanal, nonanal, (E)‐2‐nonenal, decanal, (E)‐2‐decenal, and dodecanal. The study found that appropriate fasting temporary rearing improved the odor quality of *C. reevesii* meat; OP temporary rearing for 40–50 days showed a significant reduction in greasy and musty odors. In GH, 40 days temporary rearing retained a fresh aroma while reducing the seaweed and fishy odors, achieving a flavor similar to OP. This study provides data support for *C. reevesii* meat processing and suggests a reference for applying temporary rearing to enhance the flavor quality of aquatic products.

## Graphical abstract

*Chinemys reevesii* is a special economic aquaculture species in China and is valued highly for its medicinal and nutritional benefits. This paper presents the first systematic study on the volatile odor compounds of *C. reevesii* . Pond culture and greenhouse culture are the most common aquaculture modes of *C. reevesii* . This article presents the first accurate qualitative and quantitative determination of volatile odor compounds in *C. reevesii* meat and compares the differences in odor quality between two aquaculture modes. *C. reevesii* has a slow metabolism and was subjected to temporary rearing experiments to improve its odor quality at low cost. The role of temporary rearing in enhancing the odor quality of turtles was investigated.

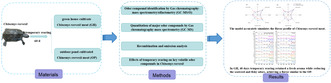

## 1 Introduction

*Chinemys reevesii* , commonly known as the Chinese grass turtle, Chinese pond turtle, and Chinese three‐keeled pond turtle, is primarily distributed in China, Korea, and Japan. The species has long been consumed as both food and medicine in East and Southeast Asia. The first Chinese pharmacopeia, the *Compendium of Materia Medica*, recorded 440 years ago, documented the significant medicinal value of *C. reevesii* . Since 2017, China's annual freshwater turtles production has been maintained at more than 45,000 tons, with farmed production reaching 53,900 tons in 2022, making China the largest consumer of *C. reevesii* globally (Ministry of Agriculture and Rural Affairs of Fisheries and Fishery Administration 2023; Xu 2017). *C. reevesii* muscle is rich in protein and has a balanced amino acid composition and a rich content of unsaturated fatty acids, especially high levels of docosahexaenoic acid (DHA) and eicosapentaenoic acid (EPA). Additionally, its muscle contains essential minerals and trace elements, among which iron, zinc, calcium, and selenium are all relatively high. It indicates that *C. reevesii* is of high nutritional value and has a health‐promoting effect (Xu 2017).

*C. reevesii* is well‐suited for captive breeding due to its strong environmental adaptability and low water quality requirements. The turtle has strong environmental adaptability and low water quality requirements, making them particularly suitable for aquaculture (Xu 2017). However, in large‐scale cultivation, uneaten feed and excrement accumulate decomposing into organic pollutants such as nitrogen and phosphorus, producing odorous substances. Moreover, microbial growth in the cultivation environment releases large quantities of odor compounds. These environmental odors are absorbed by *C. reevesii* during respiration and feeding, adversely affecting the flavor quality of the meat (McCrummen et al. 2018; Tucker and Schrader 2020). To reduce the off‐flavors in *C. reevesii* meat, complex cooking processes and various spices are often used. This not only increases the cost of turtle food processing but also is unfriendly to some people with lighter dietary habits. Accordingly, research aimed at enhancing the odor quality of *C. reevesii* meat is crucial for reducing off‐flavors, increasing consumer preference, and enhancing economic value.

Xu (2017) demonstrated that using different spices could alter the volatile odor compounds in *C. reevesii* meat, making it less fishy. However, recent research tends to focus on the use of greener, more efficient, and more convenient methods to fundamentally enhance muscle quality. Fasting temporary rearing before marketing or slaughter—a common practice in the industrial farming of fish and other aquatic animals—is an expedient quality enhancement strategy. Fasting temporary rearing allows for the alteration of microorganisms from the intestinal tract, prolonging the storage time of the meat (Suárez et al. 2010). It also changes the textural properties of the fish, increasing its springiness and hardness (Imsland et al. 2020; Zhong et al. 2018). Additionally, appropriate fasting temporary rearing can alter the nutritional profile of aquatic animals. Studies on species such as Dentex ( *Dentex dentex* ) (Suárez et al. 2010), Paddlefish ( *Polyodon spathula* ) (Nie et al. 2017), darkbarbel catfish (*Pelteobagrus vachelli*) (Qin et al. 2015), and large yellow croaker ( *Larimichthys crocea* ) (Liu et al. 2018) have demonstrated that fasting temporary rearing not only reduces fat content but also significantly increases the proportion of unsaturated fatty acids, making the fish composition healthier.

However, the most significant effect of fasting temporary rearing on the flavor quality of aquatic animals. Studies on Murray cod ( *Maccullochella peelii* ) (Palmeri et al. 2008) and Southern flounder ( *Paralichthys lethostigma* ) (Drake et al. 2010) have displayed that consumers prefer starved fish. This is because fasting temporary rearing increases the content of umami nucleotides and amino acids in aquatic animals, therefore enhancing their sweet and umami taste (Liu et al. 2018; Yang et al. 2020; Zou et al. 2023). Simultaneously, fasting temporary rearing reduces the content of total volatile base nitrogen (TVB‐N), geosmin (GSM), 2‐methylisoborneol (2‐MIB), and aldehydes and ketones with off‐flavors in aquatic animals, which significantly improves their flavor quality (Burr et al. 2012; Guo et al. 2018; Lv et al. 2018; Zou et al. 2023).

Despite extensive research on fish quality regulation through temporary rearing, studies on the effects of temporary rearing on *C. reevesii* meat odor quality are lacking. Pond culture and greenhouse culture are the most common aquacultural modes for *C. reevesii* . Therefore, this study aimed to investigate the role of temporary rearing in enhancing the odor quality of *C. reevesii* muscle in both aquacultural modes. Using GC–MS/O and sensory evaluation or odor activity value combined with aroma recombination, this research systematically identified the key volatile odor compounds in *C. reevesii* muscle and elucidated the patterns of key odor compound changes during temporary rearing.

## 2 Materials and Methods

### 2.1 Materials

Experimental Materials: Outdoor pond‐cultivated *C. reevesii* (OP) and greenhouse‐cultivated *C. reevesii* (GH), both sourced from Jiangxi Jinguiwang Industrial Co. Ltd. All *C. reevesii* used in the experiment were female and 4 years old. The culture conditions of OP before the experiment were as follows: outdoor ponds, a culture density of 8/m^2^, feeding twice a day, and 2% of body weight. Before the experiment, GH was reared in an indoor greenhouse at a constant temperature of 26°C ± 2°C, with a culture density of 8/m^2^, feeding three times a day and 2% of body weight. The average weight of the OP was 716.66 ± 45.6 g, while the GH averaged 658.75 ± 38.85 g. *C. reevesii* was transported to the laboratory at the Yangtze River Fisheries Research Institute of the Chinese Academy of Fishery Sciences and placed in cultivation tanks measuring 2 × 1 × 0.3 m with a water depth of 3 cm. The stocking density is about 8/m^2^. The water was changed daily, and no food was provided as the *C. reevesii* underwent a temporary rearing experiment (water temperature maintained at 25°C ± 2°C). Samples were taken on days 0 (control), 10, 20, 30, 40, 50, and 60 of temporary rearing. Four *C. reevesii* from OP and GH was sampled each time. The skin was removed, and the limb muscles were harvested, immediately frozen in liquid nitrogen for 3 min, and then thoroughly ground into a powder using a grinder. The resulting frozen *C. reevesii* meat powder was stored at −80°C for subsequent volatile compound analysis.

Chemicals: Dichloromethane (HPLC, 99.9%) was purchased from Avantor Performance Materials. Sodium chloride (HPLC, ≥ 99.5%), hexanol (≥ 99.5%), heptanal (≥ 98%), (E)‐2‐nonenal (≥ 95%), indole (≥ 99.5%), (E,E)‐2,4‐decadienal (≥ 90%), pentadecanal (95%), tridecanal (95.5%), hexadecanal (98%), hexanoic acid (≥ 99.5%), and nonanoic acid (≥ 99.5%) were procured from Shanghai Macklin Biochemical Co. Ltd. Decanal (≥ 98%) and nonanal (≥ 98%) were acquired from Shanghai Yuan Ye Biological Technology Co. Ltd. Hexanol (100 μg/mL) and dodecanol (100 μg/mL) were bought from Dr. Ehrenstorfer. (E)‐2‐decenal (100 μg/mL), octanal (1000 μg/mL), heptanol (100 μg/mL), tetradecanol (1000 μg/mL), γ‐nonanolactone (1000 μg/mL), and naphthalene (1000 μg/mL) were obtained from Tianjin Alta Scientific Co. Ltd. Octanol (≥ 99.5%), dodecanal (≥ 99.5%), 1‐octen‐3‐ol (≥ 99.9%), and 2‐pentylfuran (≥ 99.2%) were obtained from Beijing Tanmo Quality Inspection Technology Co. Ltd.

### 2.2 Methods

#### 2.2.1 Volatile Odor Compound Extraction Methods

To ensure the accuracy and qualitative accuracy of the results, two methods, direct solvent extraction‐solvent‐assisted flavor evaporation (DSE‐SAFE) and headspace‐solid phase microextraction (HS‐SPME), were used to extract volatile odor compounds (Guo et al. 2021).

DSE‐SAFE extraction method: 50.00 g of frozen *C. reevesii* meat powder was weighed and placed into a 500‐mL conical flask, to which 150 mL of dichloromethane was added and sealed with aluminum foil. The mixture was then extracted by shaking at 150 rpm for 2 h, and the extract was filtered and collected. This process was repeated twice more, each with an additional 100 mL of dichloromethane. The combined extracts were transferred to a SAFE device (Glasbläserei Bahr, Germany) to remove nonvolatile compounds. The conditions for SAFE were as follows: temperature at 46°C and vacuum at 9.8 × 10^−4^ Pa. Anhydrous sodium sulfate was added to the extract collected after SAFE and allowed to stand overnight. The extract was then concentrated to 1 mL using a stream of nitrogen at a micro flow rate, resulting in a concentrated odor extract from the frozen *C. reevesii* meat, which was then analyzed using the gas chromatography–mass spectrometry/olfactometry (GC–MS/O).

HS‐SPME extraction method: 2 g of frozen *C. reevesii* meat powder was accurately weighed into a 20‐mL headspace vial, in which 8 mL of saturated NaCl solution and 1 μL of 1000 mg/L cyclohexanone as an internal standard was added. An SPME fiber (50/30 μm DVB/CAR/PDMS, Sigma‐Aldrich, USA) was exposed to the headspace approximately 1 cm above the sample surface at 70°C for 30 min. The fiber was then desorbed in the injector of the GC–MS/O (Thermo, Singapore) within 3 min for 5 min.

#### 2.2.2 Gas Chromatography–Mass Spectrometry/Olfactometry

Following the methodology of An et al. (2020) analyses were conducted using the Thermo TRACE 1300 Gas Chromatography system, Thermo ISQ7000 Mass Selective Detector, and a Gerstel olfactometer. The olfactometer and mass spectrometer were set to a split ratio of 1:1 to ensure that odors detected by the olfactometer corresponded with mass spectral peaks. Volatile odor compounds were identified using the method described by An et al. (2024) with slight modification. Each sample was evaluated by at least three trained sensory analysts who recorded the retention times, odor descriptions, and odor intensities (on a scale of 0–5) for each flavor component, where 0 = no odor, 1 = very weak, 2 = slightly weak, 3 = a moderate odor, 4 = strong and 5 = a very strong odor. The GC injector temperature was set to 250°C, operated in splitless mode, with helium as the carrier gas at a flow rate of 2 mL/min. The column used was an HP‐5 column (30 m length, 0.32 mm inner diameter, and 0.25 μm film thickness). The temperature program was initiated at 40°C, held for 4 min, then ramped at 4°C/min to 250°C, and held for 10 min. Mass spectrometry conditions were set as follows: electron impact energy of 70 eV, ion source temperature of 230°C, solvent delay of 6 min, and full‐scan mode with an ion scan range of 35–350 m/z. System control and data management or analysis were performed using Xcalibur software (Thermo, USA).

#### 2.2.3 Odor Compound Identification Method

Using Xcalibur software, the mass spectra of compounds were compared with the NIST 17 database. A standard mixture of n‐alkane (C5–C30) was prepared and analyzed under the same conditions as the samples for GC–MS analysis. The retention indices (RIs) were calculated according to the Kováts method (Kovats 1958). The identification of compounds was performed by comparing the mass spectra, aroma profiles, and RIs of the compound standards.

$$\mathrm{RI}=100\times n+100\times \frac{t\left(x\right)-t\left(C_{n}\right)}{t\left(C_{n+1}\right)-t\left(C_{n}\right)}$$

where RI is the retention index, *t*(*x*) is the retention time of compound x to be identified, *t*(*C*_*n*_): the retention time for n‐alkanes with *n* carbon atoms, and *t*(*C*_*n*+1_) is the retention time for n‐alkanes with *n* + 1 carbon atoms.

#### 2.2.4 Quantitative Analysis of Major Odor Compounds

Based on GC–MS/O results, odor compounds with odor intensity ≥ 1 were selected for quantitative analysis. The HP‐SPME method was employed for quantification, using a 20‐mL headspace vial, to which 10 mL of saturated NaCl solution, 1 μL of 1000 mg/mL cyclohexanone internal standard solution, and a gradient concentration of mixed standard solutions of the compounds under investigation were added. The extraction was performed according to the method described in Section 2.2.1, and following extraction, GC–MS analysis was conducted under the same conditions as those employed for the samples.

#### 2.2.5 Determination of Odor Activity Values

Standard odor compounds were purchased, and standard curves were established under the same processing conditions as the samples to perform precise quantification of volatile odor compounds in *C. reevesii* meat. The odor activity value (OAV) is the ratio of the actual concentration of volatile odorants in *C. reevesii* meat after precise quantification to the sensory detection threshold obtained in the literature, calculated by the following formula:

$$\mathrm{OAV}=\frac{C}{\mathrm{OT}}$$

where *C* is the concentration of volatile odor compounds in *C. reevesii* meat (μg/kg), and OT is the odor threshold of the compound in water (μg/kg), obtained either through reference literature or experimental determination.

For odor thresholds unknown in the literature, the method described by An et al. (2020) was used for determination. Initially, odor compounds were dissolved in ethanol at a specified concentration, and then 10.00 μL of this odorant‐ethanol solution was added to 100 mL of pure water to create an aqueous solution of the odor compound. This solution was then serially diluted with pure water at a volume ratio of 1:3 (water solution). A 5 mL of each dilution was placed in a 20‐mL glass vial, and each dilution sample was paired with two identical vials containing 5 mL of water for a sensory triangle test. Czerny et al. (2008) described the method of odor threshold, which was calculated using each sensory analyst. The threshold of the compound is the geometric mean of the values obtained by all sensory analysts. Both the recognition odor threshold (ROT) and the detection odor threshold (DOT) are calculated, with DOT being used for the OAV calculation.

$${\mathrm{OT}}_{i}=\sqrt{C_{x}\times C_{x+1}}$$

where OT_*i*_ is the odor threshold obtained by each sensory analyst, *C*_*x*_ is the lowest concentration of odor that can be identified, *C*_*x*+1_ is the highest concentration of odor that cannot be identified.

#### 2.2.6 Recombination and Omission Analysis

An et al. (2020) described the recombination experiments in which compounds with an OAV ≥ 1 were selected and dissolved in 0.2 mol/L phosphate buffer (pH 6.8) based on their calculated concentrations and then diluted tenfold with the same buffer. *C. reevesii* meat dilutions were prepared by weighing 10 g of frozen powdered *C. reevesii* meat into 90 mL of 0.2 mol/L phosphate buffer (pH 6.8). Sensory evaluations of the diluted recombination and control samples were performed at room temperature. A total of 11 trained sensory analysts were invited to sniff and identify different aromatic characteristics (nutty, grassy or green, metallic, fresh fishy, unpleasant fishy, greasy, earthy, and seaweed‐like), rate the intensity of each aroma (0–5), and evaluate the similarity percentage between the recombination samples and the control.

Omission analysis was conducted using the method by Zhang et al. (2022) In total, 17 and 14 omission models were prepared by omitting individual compounds or aromatic standards using 0.20 mol/L phosphate buffer. All omission models were placed in 20 mL covered clear vials and equilibrated at 4°C for 48 h, and then randomly numbered before sensory evaluation. Each omission model was compared with the recombination model using a triangular test, during which 23 panel members were asked to identify the discrepant sample among the three models.

### 2.3 Data Analysis

All data analyses were performed using IBM SPSS Statistics 26 (SPSS Inc., Chicago, IL, USA). Differences between the two groups were compared using an independent samples *t*‐test, with *p* < 0.05 indicating statistical significance and *p* < 0.01 indicating highly significant differences. Analysis of variance (ANOVA) was used to compare differences among multiple groups, with *p* < 0.05 denoting statistical significance. All figures in the article were created using Origin 2023b.

## 3 Results

### 3.1 Volatile Odor Compounds in the Meat of *C. reevesii*

During the extraction of volatile odor compounds, due to different extraction principles, various methods may yield different types of detectable compounds (Guo et al. 2021). To comprehensively identify volatile odor compounds in *C. reevesii* meat from two aquacultural models, this study employed DSE‐SAFE and HS‐SPME methods combined with GC–MS/O for extensive identification. Table 1 describes a minor difference in the types of volatile odor compounds between OP and GH *C. reevesii* meat. In OP, 43 volatile compounds were detected, with 24 having an odor intensity ≥ 1, including 11 aldehydes and seven alcohols. A total of 41 volatile odor compounds were detected in GH, and 22 compounds with odor intensity ≥ 1, including 12 aldehydes and 5 alcohols. Aldehydes and alcohols are prevalent and significant in contributing to the odor profile of *C. reevesii* meat, with aldehydes showing higher odor intensity.

**TABLE 1:** Volatile odor compounds identified by gas chromatography–mass spectrometry/olfactometry (GC–MS/O).

RI	RT	CAS	Compounds	Extraction methods	Odor	Intensity	Aquacultural model
723	6.45	66‐25‐1	Hexanal	SPME, SAFE	Green, grassy	3	OP, GH
854	8.83	111‐27‐3	Hexanol	SPME	Green, musty	2	OP, GH
878	9.62	100‐42‐5	Styrene	SAFE	Floral	< 1	OP, GH
892	10.12	111‐71‐7	Heptanal	SPME, SAFE	Greasy	3	OP, GH
857	8.90	106‐42‐3	p‐Xylene	SAFE	Plastic	< 1	GH
953	12.44	100‐52‐7	Benzaldehyde	SPME, SAFE	Bitter almond	2	GH
964	12.88	111‐70‐6	Heptanol	SPME	Fatty, greasy	1	OP, GH
974	13.28	3391‐86‐4	1‐Octen‐3‐ol	SPME	Mushroom, earthy	3	OP, GH
973	13.40	142‐62‐1	Hexanoic acid	SAFE	Vinegar	1	OP, GH
987	13.74	3777‐69‐3	2‐Pentyl‐furan	SAFE	Green bean, cabbage	2	OP, GH
998	14.22	124‐13‐0	Octanal	SPME	Soapy	3	OP, GH
1027	15.22	104‐76‐7	2‐Ethylhexanol	SPME, SAFE	Fruity	< 1	OP, GH
1060	16.60	98‐86‐2	Acetophenone	SPME, SAFE	Medicine	< 1	OP
1063	16.73	26001‐58‐1	(Z)‐2‐Octen‐1‐ol	SPME	Grassy	< 1	OP, GH
1068	16.90	111‐87‐5	Octanol	SPME, SAFE	Fatty, leafy	2	OP, GH
1094	17.89	2277‐16‐9	(E)‐4‐Nonenal	SAFE	Fruity	< 1	OP, GH
1102	18.22	124‐19‐6	Nonanal	SPME, SAFE	Fatty	4	OP, GH
1144	19.78	76‐22‐2	Camphor	SPME	Medicine	< 1	OP, GH
1151	20.04	10340‐23‐5	(Z)‐3‐Nonen‐1‐ol	SPME	Mushroom	< 1	OP, GH
1157	20.29	18829‐56‐6	(E)‐2‐Nonenal	SPME, SAFE	Fatty	3	OP, GH
1169	20.72	143‐08‐8	Nonanol	SPME	Citrus	< 1	OP, GH
1171	20.78	124‐07‐2	Octanoic acid	SAFE	Sour, rancid	< 1	OP, GH
1180	21.14	91‐20‐3	Naphthalene	SPME, SAFE	Metallic	2	OP
1204	22.01	112‐31‐2	Decanal	SPME, SAFE	Oily	4	OP, GH
1220	22.60	95‐16‐9	Benzothiazole	SPME, SAFE	Floral	< 1	OP, GH
1237	23.41	927‐49‐1	6‐Undecanone	SAFE	Fruity	< 1	OP, GH
1263	24.05	3913‐81‐3	(E)‐2‐Decenal	SAFE	Fatty, fishy	5	OP, GH
1267	24.24	112‐05‐0	Nonanoic acid	SAFE	Fatty, waxy	1	OP, GH
1288	24.98	120‐72‐9	Indole	SPME, SAFE	Nutty	2	OP, GH
1305	25.58	112‐44‐7	Undecanal	SPME, SAFE	Citrus	< 1	OP, GH
1318	25.98	25152‐84‐5	(E,E)‐2,4‐Decanedienal	SAFE	Fishy, river water	5	OP, GH
1357	27.24	104‐61‐0	γ‐Nonanolactone	SPME, SAFE	Coconut	2	OP, GH
1371	27.82	112‐42‐5	Undecanol	SAFE	Citrus, fatty	1	OP, GH
1365	27.55	334‐48‐5	Decanoic acid	SAFE	Sour	< 1	OP, GH
1407	28.94	112‐54‐9	Dodecanal	SPME	Leafy, fatty	2	OP, GH
1446	30.12	3796‐70‐1	Geranylacetone	SPME, SAFE	Floral, milky	< 1	OP, GH
1471	30.97	112‐53‐8	Dodecanol	SPME, SAFE	Citrus	< 1	OP, GH
1510	32.18	10486‐19‐8	Tridecanal	SAFE	Soapy	< 1	OP, GH
1558	33.60	143‐07‐7	Dodecanoic acid	SAFE	Fatty	< 1	OP, GH
1606	35.00	28231‐03‐0	Cedrenol	SPME, SAFE	Woody	2	OP
1676	36.92	112‐72‐1	Tetradecanol	SPME	Fatty	1	OP
1714	37.98	2765‐11‐9	Pentadecanal	SPME, SAFE	Soapy	1	OP, GH
1758	39.23	544‐63‐8	Myristic acid	SAFE	Fatty, vinegar	< 1	OP, GH
1813	40.69	629‐80‐1	Hexadecanal	SPME, SAFE	Leafy, fatty	2	OP, GH
1936	43.79	373‐49‐9	Palmitoleic acid	SAFE	Fatty	< 1	OP, GH

### 3.2 Content and OAV of Volatile Odor Compounds in *C. reevesii* Meat

In the identification using GC–MS/O in Section 3.1, odor intensity descriptions were provided by three sensory panel members. Compounds with an odor intensity of < 1, perceived by only one or two members, indicate extremely weak odors or differences among sensory personnel. To ensure accuracy, only 25 compounds with an odor intensity of at least 1 perceived by all three members were quantitatively analyzed. Table 2 presents the OAV of these compounds in *C. reevesii* meat. Compounds such as benzaldehyde, hexanoic acid, and undecanol, although present in high contents, have high thresholds, resulting in an OAV < 1, making them minor contributors to *C. reevesii* meat flavor. Compounds with an OAV ≥ 1 are considered significant contributors to the overall flavor profile.

**TABLE 2:** Internal standards, target ions, calibration equations, injection concentration ranges, and *R* ^2^ for major aroma compounds.

Compounds	Qualitative ions	Quantitative ions	Injection range (μg/L)	Equation	*R* ^2^	Thresholds (μg/kg)	OAV	
							OP	GH
Cyclohexanone^a^	42, 55, 98	55						
Hexanal	41, 44, 56	41	1–20	y = 0.0043x + 0.0108	0.998	2.4 (Mall and Schieberle 2017)	23	41
Hexanol	41, 43, 56	56	1–20	y = 0.0063x + 0.0139	0.999	5.6 (Van Gemert 2011)	2	4
Heptanal	41, 44, 70	70	1–20	y = 0.0122x − 0.0044	0.995	2.8 (Van Gemert 2011)	4	3
Benzaldehyde	77, 105, 106	77	1–50	y = 0.0672x + 0.0675	0.996	751 (Van Gemert 2011)	< 1	< 1
Heptanol	43, 56, 70	70	1–20	y = 0.0247x − 0.0045	0.996	5.4 (Van Gemert 2011)	1	1
Hexanoic acid	41, 60, 73	73	1–20	y = 0.0002x + 0.0004	0.995	890 (Van Gemert 2011)	< 1	< 1
1‐Octen‐3‐ol	43, 57, 72	57	1–50	y = 0.0912x − 0.3371	0.999	1.2 (Frank et al. 2009)	21	24
2‐Pentyl‐ furan	81, 138	81	0.08–2	y = 0.0147x − 0.001	0.998	5.8 (Van Gemert 2011)	1	1
Octanal	43, 44, 56	43	1–20	y = 0.0369x − 0.2697	0.998	3.4 (Mall and Schieberle 2017)	2	4
Octanol	41, 55, 56	56	1–20	y = 0.0929x − 0.9033	0.998	125.8 (Van Gemert 2011)	< 1	< 1
Nonanal	41, 43, 57	57	1–50	y = 0.0104x + 0.1969	0.999	2.8 (Mall and Schieberle 2017)	21	8
(E)‐2‐Nonenal	43, 55, 70	55	0.08–2	y = 0.0134x − 0.0057	0.998	0.19 (Mall and Schieberle 2017)	6	3
Naphthalene	102, 128	128	1–50	y = 0.466x − 10.601	0.995	6 (Van Gemert 2011)	2	—
Decanal	41, 55, 57	57	1–50	y = 0.0098x − 0.1206	0.999	3 (Van Gemert 2011)	10	3
Nonanoic acid	57, 60, 73	60	1–20	y = 0.0002x + 0.0012	0.999	4600 (Van Gemert 2011)	< 1	< 1
(E)‐2‐Decenal	43, 55, 70	70	1–50	y = 0.0761x − 1.2752	0.999	0.35 (Van Gemert 2011)	515	44
Indole	70, 117	117	1–20	y = 0.0137x − 0.1033	0.997	40 (Van Gemert 2011)	< 1	< 1
(E,E)‐2,4‐Decanedienal	41, 81	81	0.08–2	y = 0.0342x − 0.0031	0.999	0.027 (Van Gemert 2011)	243	195
γ‐Nonanolactone	85	85	1–20	y = 0.0614x − 0.8004	0.994	9.7 (Van Gemert 2011)	2	< 1
Undecanol	41, 55, 69	55	1–20	y = 0.0284x − 0.3759	0.996	700 (Van Gemert 2011)	< 1	< 1
Dodecanal	41, 55, 57	57	1–50	y = 0.0086x − 0.0552	0.999	0.29 (Van Gemert 2011)	220	46
Dodecanol	43, 55, 69	69	1–50	y = 0.0682x − 0.5851	0.998	16 (Van Gemert 2011)	< 1	< 1
Tridecanal	43, 57, 82	57	1–20	y = 0.0303x − 0.2657	0.999	10 (Van Gemert 2011)	< 1	< 1
Cedrenol	43, 95, 150	95	1–20	y = 0.0111x − 0.0761	0.997	122.07^b^	< 1	—
Tetradecanol	43, 55, 69	69	1–50	y = 0.0306x − 0.0239	0.998	3.052^b^	1	—
Pentadecanal	43, 57, 82	82	1–20	y = 0.028x − 0.3478	0.999	1000 (Van Gemert 2011)	< 1	< 1
Hexadecanal	43, 57, 82	82	1–20	y = 0.0242x − 0.2717	0.999	0.91 (Xu et al. 2023)	7	8

^a^ Is an internal standard.

^b^ Odor thresholds are measured in the laboratory.

In OP, 17 volatile compounds had an OAV ≥ 1, with three exceeding 100: (E)‐2‐decenal (OAV = 515), (E,E)‐2,4‐decanedienal (OAV = 243), and dodecanal (OAV = 220), contributing oily, fishy, and leafy. Compounds with an OAV between 10 and 100 include hexanal (OAV = 23), 1‐octen‐3‐ol (OAV = 21), nonanal (OAV = 21), and decanal (OAV = 10), providing grassy and mushroom or earthy. GH shows differences in the types and quantities of volatile odor compounds compared to OP, lacking naphthalene, cedrenol, and tetradecanol observed in OP. In GH, 14 compounds had an OAV ≥ 1, with only (E,E)‐2,4‐decanedienal (OAV = 195) exceeding 100. Compounds with an OAV between 10 and 100 include hexanal (OAV = 41), 1‐octen‐3‐ol (OAV = 24), (E)‐2‐decenal (OAV = 44), and dodecanal (OAV = 44), contributing to the distinctive aroma differences between the two aquaculture models.

### 3.3 Recombination and Omission Analysis

**FIGURE 1:**
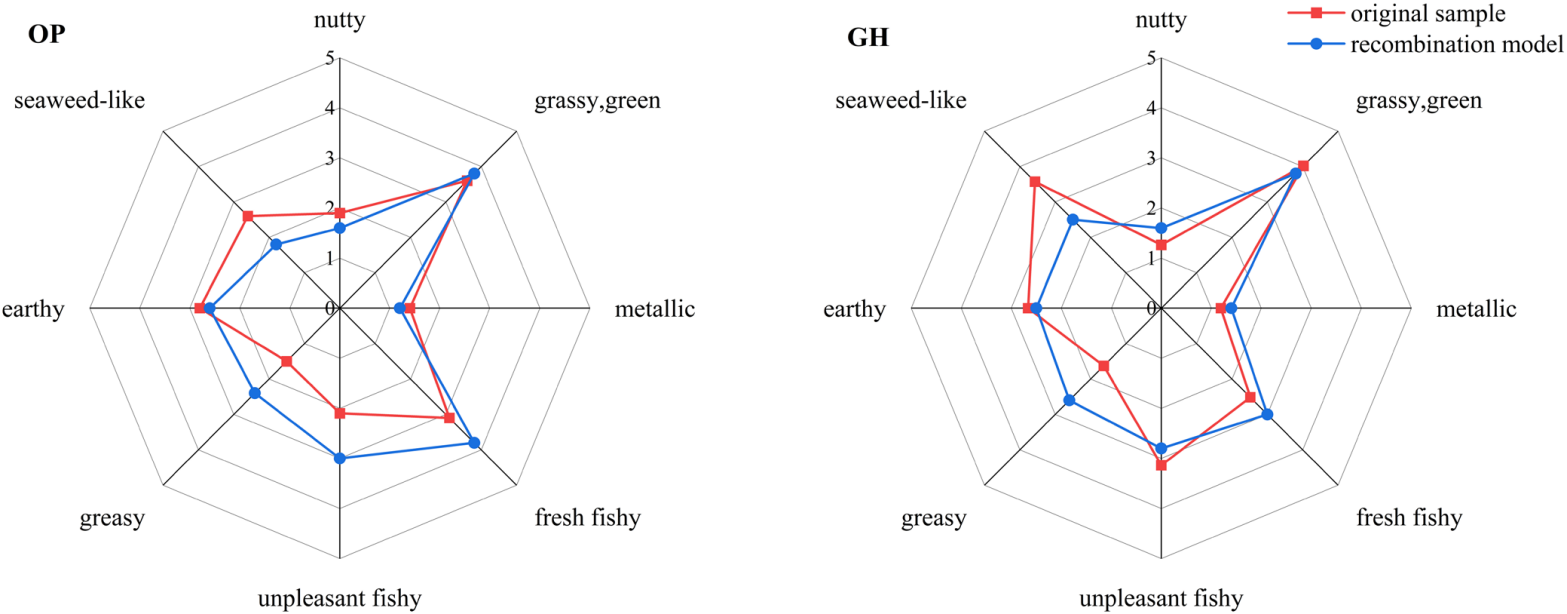
Aroma profiles of original samples and recombination model of *Chinemys reevesii* .

To verify the accuracy of qualitative and quantitative results of odor compounds in *C. reevesii* meat, recombination experiments were conducted on compounds with an OAV ≥ 1 (Table 2), and sensory evaluations of these models with results are depicted in Figure 1. The *t*‐test comparing sensory scores between the recombination model and *C. reevesii* meat samples demonstrated an insignificant difference between the two aquaculture models (*p* > 0.05), indicating that the model accurately simulates the flavor profile of *C. reevesii* meat. This suggests that the qualitative and quantitative results for odor compounds in this study are reliable for evaluating their odor characteristics. Differences between recombination and original samples were primarily noted in seaweed‐like, greasy, and unpleasant fishy odors, likely due to masking, inhibitory, or synergistic effects among compounds with OAV < 1 and those with OAV > 1 (Zhang et al. 2022). It can be observed from the sensory description that the different types and contents of volatile odor compounds exhibit the odor of *C. reevesii* meat in the two aquaculture models differently. The GH has a stronger seaweed‐like and fishy odor than the OP, making the *C. reevesii* meat cultured in the greenhouse have poor flavor, but the thicker, greasy odor in the *C. reevesii* meat cultured in the pond will also make it present a peculiar odor.

Omission experiments were conducted to study the contributions of specific compounds to the overall odor of *C. reevesii* muscle. According to Table 2, 17 and 14 omission models were prepared, each omitting a single compound for comparison against the recombination models. Table 3 presents OP and GH demonstrated significant differences in 10 models in the omitted experiments (*p* < 0.05). Significant differences were observed in OP when hexanal, heptanal, 1‐octen‐3‐ol, octanal, nonanal, (E)‐2‐nonenal, decanal, (E)‐2‐decenal, (E,E)‐2,4‐decadienal, and dodecanal were omitted. In GH, significant differences occurred upon omission of hexanal, hexanol, heptanal, 1‐octen‐3‐ol, octanal, nonanal, (E)‐2‐nonenal, decanal, (E)‐2‐decenal, and dodecanal. These are key odor compounds for OP and GH.

**TABLE 3:** Influence of aroma compounds on the overall aroma profiles.

Compounds omitted	OP		GH	
	*n*/23	Significance	*n*/23	Significance
Hexanal	18	*	19	*
Hexanol	7	ns	15	*
Heptanal	18	*	18	*
Heptanol	4	ns	2	ns
1‐Octen‐3‐ol	20	*	19	*
2‐Pentyl‐ furan	3	ns	4	ns
Octanal	12	*	16	*
Nonanal	17	*	18	*
(E)‐2‐Nonenal	19	*	16	*
Naphthalene	5	ns	—	—
Decanal	16	*	17	*
(E)‐2‐Decenal	18	*	18	*
(E,E)‐2,4‐Decanedienal	16	*	7	ns
γ‐Nonanolactone	4	ns	—	—
Dodecanal	15	*	15	*
Tetradecanol	9	ns	—	—
Hexadecanal	5	ns	6	ns

*Note: n* means the number of panelists who perceived differences in the triangle test correctly.

* Means *p* < 0.05, with significance; ns is not significant.

Additionally, the seven compounds removed from OP with OAV < 10, but heptanal (OAV = 4) and octanal (OAV = 2) were omitted to indicate significant differences, whereas (E,E)‐2,4‐decadienal (OAV = 195) was omitted from GH indicating insignificant differences. This suggests that OAV was unaffected by the significance of results in omission experiments and cannot be the sole criterion for identifying key odor compounds in *C. reevesii* meat, consistent with the findings of Zhang et al. (2022).

### 3.4 Effects of Temporary Rearing on Key Volatile Odor Compounds in *C. reevesii*

**FIGURE 2:**
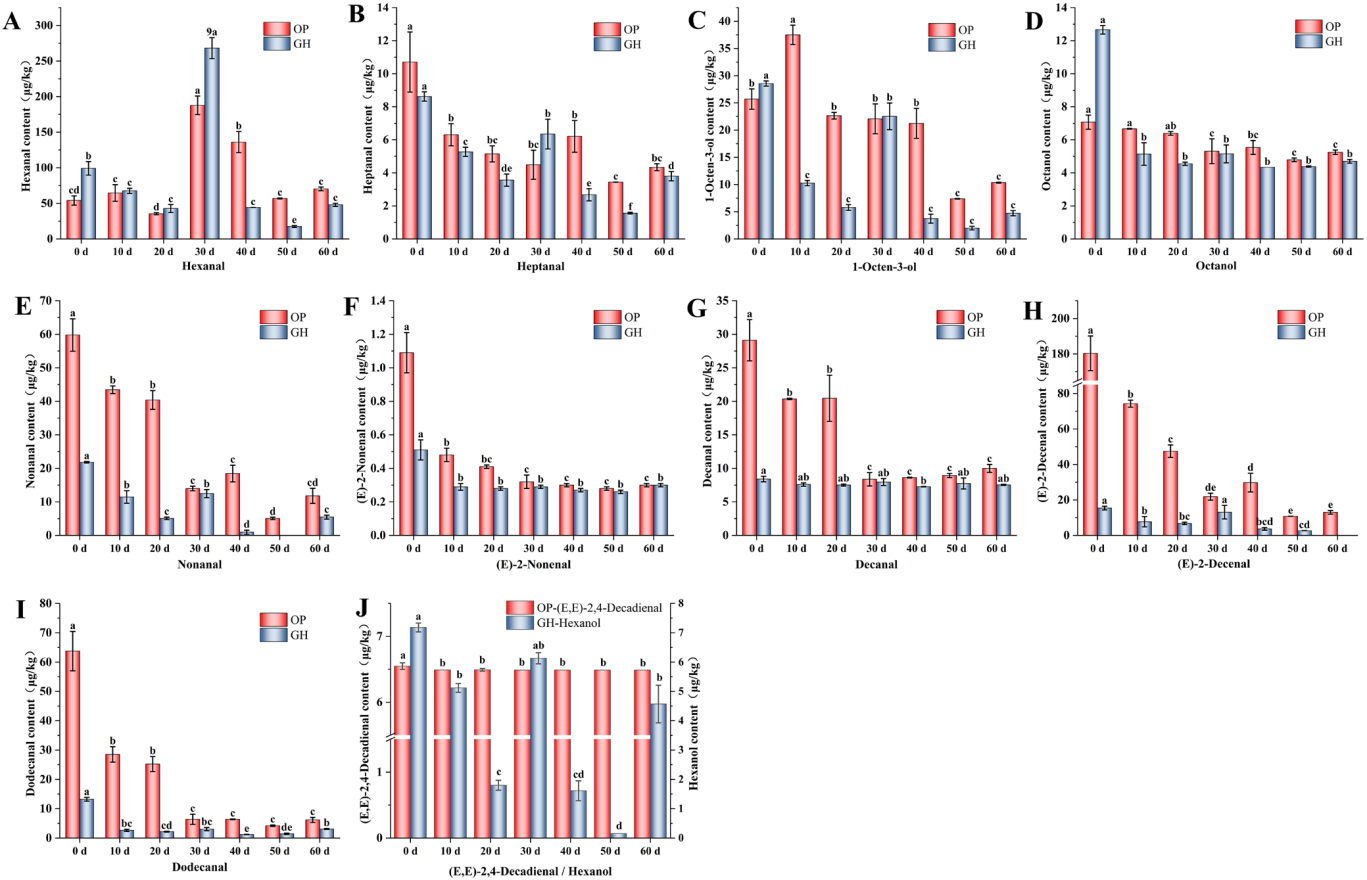
Effect of temporary rearing on the content of key volatile odor compounds in *Chinemys reevesii* cultured in outdoor pond (OP) and greenhouse (GH), including hexanal content (A), heptanal content (B), 1‐octen‐3‐ol content (C), octanal content (D), nonanal content (E), (E)‐2‐nonenal nonanal content (F), decanal content (G), (E)‐2‐decenal content (H), dodecanal content (I), and (E,E)‐2,4‐decadienal and hexanol content (J). Different letters in the same aquacultural model indicate significant differences between days of temporary rearing (*p* < 0.05).

The impact of temporary rearing on the content of key volatile compounds in OP is depicted in Figure 2. Concentrations of heptanal, 1‐octen‐3‐ol, octanal, nonanal, (E)‐2‐nonenal, (E)‐2‐decenal, and dodecanal dropped to their lowest level after 50 days of temporary rearing. The concentration of hexanal initially decreased before increasing, reaching 187.76 μg/kg at 30 days, significantly higher than other groups. Meanwhile, at 30 days, the concentrations of heptanal, octanal, (E)‐2‐nonenal, decanal, (E)‐2‐decenal, and dodecanal were significantly lower than at 0 day, with insignificant differences between 40 and 60 days. (E,E)‐2,4‐decadienal, described as having a fishy and river water odor in *C. reevesii* meat, significantly decreased after 10 days of temporary rearing. The subsequent changes in this compound were less noticeable than those in other volatile odor compounds, necessitating further investigation.

**FIGURE 3:**
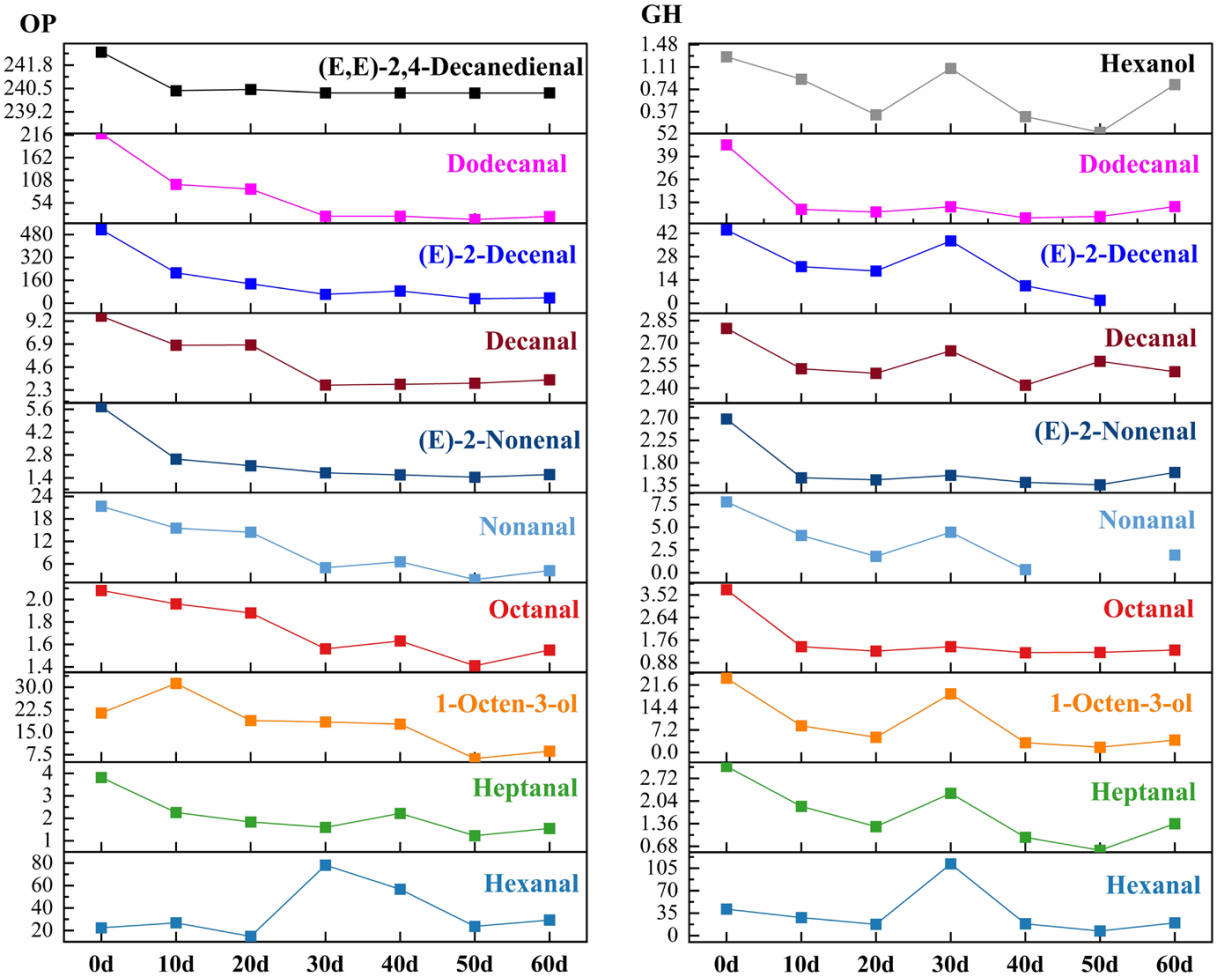
Effects of temporary rearing on the OAV of key volatile odor compounds in *Chinemys reevesii* cultured in an outdoor pond (OP) and greenhouse (GH).

After OAV analysis, compounds contributing significantly in the OP included (E)‐2‐decenal, (E,E)‐2,4‐decadienal, and dodecanal (Figure 3). The lowest OAV of these compounds were found on day 50, thus presenting the lowest contribution to the greasy and soapy odors in *C. reevesii* meat. Additionally, the OAV of 1‐octen‐3‐ol decreased from 21 to 6 at 50 days, reducing the earthy odor in OP *C. reevesii* meat. Between 30 and 60 days, OAV changes for heptanal, octanal, (E)‐2‐nonenal, decanal, and (E,E)‐2,4‐decadienal were minor, affecting the *C. reevesii* muscle flavor minimally. Therefore, to prevent an excessive concentration of hexanal from interacting with other substances and causing off‐flavors at 30 days, it is advisable to reduce the fatty and earthy odors in OP during 40–50 days of temporary rearing, resulting in a more favorable flavor profile.

The trend of hexanal content in GH after temporary rearing treatment was similar to that of OP, and both reached the highest value at 30 days of temporary rearing. The content reached 268.09 μg/kg, which was significantly higher than that of other temporary rearing groups. There was an insignificant difference in the contents of octanal, (E)‐2‐nonanal, and decanal within 10–60 days of temporary rearing, but they were significantly lower than 0 day. After 50 days of temporary rearing, the concentrations of hexanal, hexanol, heptanal, 1‐octen‐3‐ol, and (E)‐2‐nonenal reached their lowest, with nonanal below the detection limit at 50 days and (E)‐2‐decenal below the detection limit at 60 days.

Due to lower volatile odor compounds contents in GH, hexanol, heptanal, and nonanal showed OAV < 1 after temporary rearing, becoming minor contributors to the odor. Significant reductions in heptanal, 1‐octen‐3‐ol, nonanal, and dodecanal at 40 days reduced the earthy and greasy odors in GH, enhancing its odor quality. Despite nonanal and (E)‐2‐decenal being described as contributors to fatty and fishy odors in *C. reevesii* meat, the overall odor is a composite result of various odor compounds, and the absence of key volatile odor compounds can alter the overall odor profile. Accordingly, to mitigate the impact of missing odor compounds on the aroma characteristics of GH, reducing fishy and earthy odors formed by hexanol, 1‐octen‐3‐ol, and (E)‐2‐decenal after 40 days of temporary rearing can help achieve an odor similar to that in OP.

## 4 Discussion

### 4.1 Extraction of Volatile Odor Compounds

The extraction of odor compounds from aquatic animals is a critical step in the study of volatile odor compounds, where different extraction methods can lead to variations in the results (Guo et al. 2021). HS‐SPME and DSE‐SAFE are two commonly used methods in the analysis of aquatic product flavors. HS‐SPME combines collection, separation, and concentration and is known for its simplicity, cost‐effectiveness, and ease of implementation. However, its extraction often occurs under heating conditions (30°C–80°C), where high temperatures can reduce the distribution coefficient of analytes between the coating and matrix, decreasing the adsorption of analytes on the coating and affecting the sensitivity of SPME. Additionally, various volatile odor compounds are thermally unstable; high extraction temperatures can induce chemical changes, leading to distorted analytical results. Decomposition of the coating at high temperatures can also release silicon‐containing substances that interfere with GC–MS analysis of odor compounds (An 2020).

The DSE‐SAFE prevents the formation of substances unoriginally present in the sample and the degradation of odor compounds during extraction, allowing for a complete separation of complex food matrices (Salum, Guclu, and Selli 2017). However, despite the higher extraction efficiency of DSE‐SAFE for sulfur‐containing substances, the formyl groups of aldehydes and carbonyl groups of ketones are unstable and may be oxidized or reduced by organic solvents during extraction. Aldehydes, being highly volatile, can be lost during concentration by nitrogen blowing (Zhou et al. 2019). This might explain why octanal and dodecanal were only detected during HS‐SPME extraction in this study. Compared to DSE‐SAFE, HS‐SPME does unreadily absorb sulfur‐containing substances but has a higher extraction efficiency for aldehydes, alcohols, and ketones. Alcohols, formed by the reduction of aldehydes by dehydrogenases, such as the reduction of (E)‐2‐pentenal to 1‐penten‐3‐ol and (E)‐2‐hexenal to (Z)‐3‐hexen‐1‐ol, result in a higher detection of alcohols by SPME (Bezysov, Dubova, and Rogova 2015).

Accordingly, to precisely identify volatile odor compounds in *C. reevesii* meat, both methods were employed in this study, revealing that DSE‐SAFE detected 35 odor‐active volatile compounds, while HS‐SPME detected 29. Studies on fish sauce (Lapsongphon, Yongsawatdigul, and Cadwallader 2015) and salmon (Guo et al. 2021) have also shown that using both methods together achieves more accurate qualitative results.

### 4.2 Volatile Odor Compounds in *C. reevesii*

The results indicate that aldehydes and alcohols are prevalent in *C. reevesii* meat, similar to findings in black carp ( *Mylopharyngodon piceus* ), grass carp (*Ctenopharyngodon idellus*) (Shi et al. 2013), silver carp ( *Hypophthalmichthys molitrix* ) (Geng et al. 2022), and bighead carp (*Aristichthys nobilis*) (Xiao et al. 2021). In aquatic products, nonaromatic aldehydes and alcohols mostly arise from the oxidation of fatty acids and are described as contributing grassy, oily, citrus, and mushroom flavors, key to the characteristic flavors of aquatic products (An et al. 2020).

Additionally, besides aldehydes and alcohols, five other compounds with odor intensities ≥ 1 were identified in *C. reevesii* meat: hexanoic acid, nonanoic acid, 2‐pentylfuran, naphthalene, and indole. Acids such as hexanoic and nonanoic are rarely identified in fresh aquatic products but are found in fresh silver carp, cod surimi (An et al. 2020), and rainbow trout (Cengiz et al. 2023), and often in fermented or processed products such as fermented sour fish (Zhang et al. 2022), stinky mandarin fish (Li et al. 2013), and salted large yellow croaker ( *Larimichthys crocea* ) (Wei et al. 2023), typically described as sour and fatty. 2‐Pentylfuran, possibly resulting from carbohydrate dehydration or the Amadori rearrangement, is described as fresh in steamed Chinese mitten crab (Gu et al. 2014) and oyster sauce (Yu et al. 2022) but as green pea‐like and spicy in fish sauce (Giri et al. 2010), similar to this study. Naphthalene was identified as a paint odor in this study and was rarely detected in aquatic products. Consequently, it is hypothesized that it results from the same source as aromatic hydrocarbons such as toluene and p‐xylene, which are derived from the pollution of the aquaculture environment (Zhao et al. 2015).

### 4.3 Effect of Aquaculture on Volatile Odor Compounds in *C. reevesii*

As demonstrated in this study, the aquaculture environment significantly impacts odor quality, where differences in volatile odor compounds between OP and GH led to distinct odor profiles. Similar findings have been observed in studies on other aquatic products such as bighead carp (Hu et al. 2021), common carp ( *Cyprinus carpio* ) (Zou et al. 2023), barramundi ( *Lates calcarifer* ) (Frank et al. 2009), and gilthead seabream ( *Sparus aurata* ) (Grigorakis, Taylor, and Alexis 2003). The factors causing differences in odor compounds under different aquaculture conditions are manifold. In studies on Yellow River carp (*Cyprinus carpio haematopterus*) (Wang et al. 2022) and greater amberjack ( *Seriola dumerili* ) (Alexi et al. 2020), flavor differences were attributed to variations in muscle fat content, traceable to differences in feed fat source.

However, other studies suggest that water quality during aquaculture plays a major role. Zhang et al. (2021) found that nitrates and nitrites in the water are major factors affecting flavor quality. Fish in water with higher levels of nitrite and nitrate had higher levels of earthy odor compounds. The volatile odor compounds content of largemouth bass shows a negative correlation with total nitrogen and phosphorus levels in the water (Zheng et al. 2022). Additionally, microorganisms in the aquatic environment, such as cyanobacteria, microcystis, and actinomycetes, including streptomyces, which produce GSM and 2‐MIB, also affect the odor of aquatic animals. These organisms promote the synthesis of β‐cyclocitral and accumulate volatile off‐flavors in aquatic animals during respiration and feeding, degrading odor quality (Kong et al. 2012; McCrummen et al. 2018; Vidal et al. 2016).

### 4.4 Effect of Temporary Rearing on Volatile Odor Compounds in *C. reevesii*

Fasting temporary rearing in this experiment can improve the odor of *C. reevesii* meat by reducing earthy, greasy, and fishy odors. Studies display that more than 20 days of starvation in grass carp effectively reduces the content of hexanal, 1‐octen‐3‐ol, and nonanal, thereby diminishing the earthy odor in the meat, though due to nutritional and textural limitations, starvation should not exceed 50 days (Lv et al. 2018). Similar results were observed with yellow catfish starvation over 20–40 days, reducing aldehyde and ketone content related to off‐flavors (Zou et al. 2023). However, short‐term 7 days starvation in crucian carp can effectively regulate flavor, reducing off‐flavor compound content, potentially due to differences in fatty acid metabolism rates among different aquatic species (Jiang et al. 2017).

Moreover, Zou et al. (2023) found that the composition of the gut microbiota during starvation was a key factor affecting off‐flavor compound content. Starvation can adjust the diversity, community structure, and function of gut microbes, reducing GSM and 2‐MIB content and mitigating the off‐flavor in largemouth bass (Zou et al. 2023). This study did not compare gut microbiota in *C. reevesii* , presenting a new direction for future research into the effects of temporary rearing on volatile odor compounds in aquatic animals.

## 5 Conclusions

The combined use of DSE‐SAFE, HS‐SPME, GC–MS/O, and flavor recombination experiments effectively identified key volatile odor components in *C. reevesii* meat. Key volatile compounds in OP *C. reevesii* meat included hexanal, heptanal, 1‐octen‐3‐ol, octanal, nonanal, (E)‐2‐nonenal, decanal, (E)‐2‐decenal, (E,E)‐2,4‐decadienal, and dodecanal. GH *Chinemys reevesii* muscles were hexanal, hexanol, heptanal, 1‐octen‐3‐ol, octanal, nonenal, (E)‐2‐nonenal, decanal, (E)‐2‐decenal and dodecanal. Recombination experiments validated the accuracy of qualitative and quantitative results. Temporary rearing positively influenced the odor of *C. reevesii* meat; 40–50 days of temporary rearing in OP not only preserved fresh odor but also reduced greasy and earthy odors, enhancing the flavor profile. Temporary rearing for 40 days in GH similarly reduced fishy and off‐odors, achieving a flavor profile comparable to that of OP.

## Author Contributions

**Xiaorong Lu:** conceptualization (lead), data curation (equal), formal analysis (equal), investigation (lead), methodology (equal), writing – original draft (equal), writing – review and editing (equal). **Yali Yu:** methodology (equal), project administration (equal), supervision (lead), writing – original draft (equal). **Lixue Dong:** data curation (supporting), validation (equal). **Gang He:** data curation (equal), validation (equal). **Lang Zhang:** visualization (equal), writing – original draft (supporting), writing – review and editing (supporting). **Tao Mao:** visualization (equal), writing – original draft (supporting), writing – review and editing (supporting). **Yun Liu:** software (equal). **Yuntao Zhou:** funding acquisition (equal), resources (equal), writing – review and editing (equal). **Li He:** investigation (equal), resources (equal), writing – review and editing (equal).

## Ethics Statement

This experiment has passed the ethical review of experimental animals, and the relevant materials passed the review have been uploaded.

## Conflicts of Interest

The authors declare no conflicts of interest.

## Data Availability

Data will be provided by the corresponding author upon request.
